# Breast cancer cell-derived exosomes and macrophage polarization are associated with lymph node metastasis

**DOI:** 10.18632/oncotarget.23238

**Published:** 2017-12-13

**Authors:** Yin Ji Piao, Hoe Suk Kim, Eun Hye Hwang, Jisu Woo, Meihua Zhang, Woo Kyung Moon

**Affiliations:** ^1^ Department of Radiology, Seoul National University Hospital and Seoul National University College of Medicine, Jongno-Gu, Seoul 03080, Korea; ^2^ Department of Biomedical Sciences, Seoul National University College of Medicine, Jongno-Gu, Seoul 03080, Korea; ^3^ Department of Radiology, Yanbian University Hospital, Yanji City, Jilin Province 133000, China

**Keywords:** exosome, triple-negative breast cancer, lymph node, metastasis, macrophage

## Abstract

Crosstalk between breast cancer and macrophages has potential implications for tumor metastasis. This study investigates macrophage polarization induced by triple-negative breast cancer (TNBC) cell-derived exosomes that promote lymph node (LN) metastasis in orthotopic TNBC models. The MDA-MB-231 cancer cell line expressing the exosomal CD63-red fluorescence (RFP) fusion protein was generated to noninvasively visualize exosome transfer into cancer cells and macrophages. Administration of RFP-tagged exosomes enhanced migration of macrophages and induced macrophage polarization *in vitro*. In orthotopic TNBC models, noninvasive bioluminescent imaging, ultrasound-guided photoacoustic imaging, and histological analysis revealed that intravenous injection of RFP-tagged exosomes promoted primary tumor growth and axillary LN metastasis in which expression of CD206, a marker or alternatively activated type 2 (M2) macrophages, was significantly higher than expression of NOS2, a marker of classically activated type 1 (M1) macrophages. These results suggest breast cancer cell-derived exosomes stimulate macrophage polarization that creates favorable conditions for LN metastatic processes in TNBC.

## INTRODUCTION

Axillary lymph node (LN) status, one of the first signs of metastatic spread, is an independent prognostic factor for all subtypes of breast cancer [1]. Triple-negative breast cancer (TNBC), which is characterized by the absence of the estrogen (ER) and progesterone (PR) receptors and the human epidermal growth factor receptor 2 (HER2), is the most aggressive breast cancer subtype and has a poor prognosis [2]. Evidences from previous clinical studies indicate that axillary LN metastasis develops in 50% of TNBC patients, and 70% to 80% of breast cancer patients with LN metastasis experience recurrence or distant metastasis develops [3, 4]. However, the underlying mechanisms of the LN metastatic process of TNBC remain to be further explored.

Exosomes are extracellular vesicles released from various cell types and contain numerous molecular constituents of the original cells, including proteins and nucleic acids. Exosomes facilitate disease progression and cell-to-cell communication [5]. Cancer cells secrete a large number of exosomes compared with non-transformed cells [6]. Exosomes released from cancer cells stimulate cancer cell growth and mobility and the immune cell response in promoting cancer progression and metastasis [7, 8]. The pathological function of cancer-derived exosomes in cancer progression and metastasis includes modifying the immune cell response at both local and distant sites.

Macrophage polarization is a process by which macrophages express different functions in response to microenvironment signals and is a factor in tumor-suppressive or tumor-promoting immunity [9]. In the primary tumor and metastatic sites, tumor-associated macrophages are the most abundant immune cells. Macrophages exhibit different phenotypes, including classically activated macrophages (M1) or alternatively activated macrophages (M2), depending on the tumor type and stromal interactions [10, 11]. M1-type macrophages are inflammatory or anti-tumorigenic, based on the expression of inducible NO synthase (NOS2), whereas M2-type macrophages are anti-inflammatory and pro-tumorigenic based on the increased expression of CD206 and arginase-1 [12]. An increase in M2-type macrophages is a prognostic marker for poor prognosis and metastasis in diverse cancer types [13–15]. Macrophages exhibit different properties in different subtypes of breast cancer. Macrophages activated by co-culture with TNBC cells upregulate CD206, a commonly used marker of M2-type macrophages, compared with cells activated by ER breast cancer cells [16], suggesting that TNBC-exposed macrophages are more likely to exhibit M2 properties.

Studies suggest that cancer-derived exosomes can reprogram the macrophage phenotype to provide a favorable microenvironment for tumor growth and dissemination in diverse cancers [17–22]. However, little is known about the relation among cancer-derived exosome, macrophage polarization, and LN metastasis in TNBC. We generated TNBC cells that produced exosomes tagged with a red fluorescence protein (RFP) reporter gene to visualize and track cancer exosomes, and we used noninvasive bioluminescent imaging (BLI) and ultrasound (US)-guided photoacoustic imaging (PAI) to monitor tumor growth and axillary LN metastasis in orthotopic TNBC models. We investigated whether TNBC exosome-related macrophage polarization promotes LN metastasis.

## RESULTS

### Establishment of MDA-MB-231 cells expressing CD63-RFP and analysis of RFP-tagged exosomes

**Figure 1:**
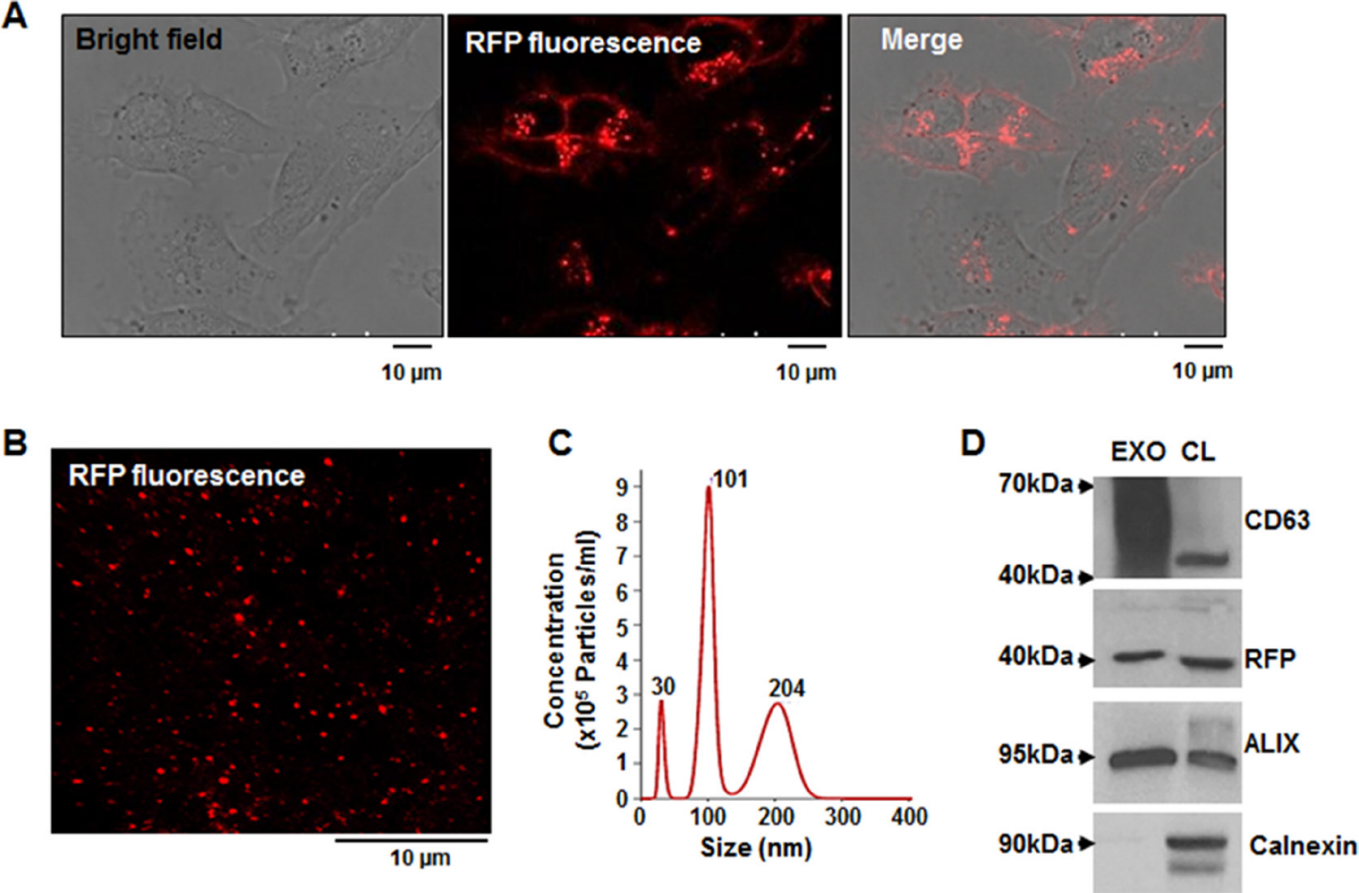
Generation of stable MDA-MB-231 cells overexpressing the exosomal CD63-RFP fusion protein and analysis of purified RFP-tagged exosomes (**A**) Confocal images of CD63-RFP–transduced MDA-MB-231 cells. (**B**) Confocal image of purified RFP-tagged exosomes. (**C**) NanoSight analysis of the size and concentration of purified RFP-tagged exosomes. (**D**) Western blot of CD63, ALIX, calnexin, and RFP in the purified RFP-tagged exosome (EXO) and the lysates of MDA-MB-231/CD63-RFP cells (CL).

The tetraspanin CD63 protein is a common exosomal biomarker. To directly image breast cancer–derived exosomes, we established a MDA-MB-231/CD63-RFP cell line that stably expresses CD63-RFP protein (Figure 1A). Confocal fluorescence images revealed RFP-tagged exosomes purified from culture supernatant of MDA-MB-231/CD63-RFP cells (Figure 1B). NanoSight results revealed that MDA-MB-231/CD63-RFP cells released exosomes with heterogeneous sizes ranging from 3 to 200 nm in diameter (Figure 1C). Western blot revealed that purified exosomes exhibited high expression of specific exosomal marker proteins, such CD63 and ALIX, but not the endoplasmic reticulum membrane marker calnexin (Figure 1D).

### TNBC cell–derived exosomes promote TNBC cell migration and proliferation

**Figure 2:**
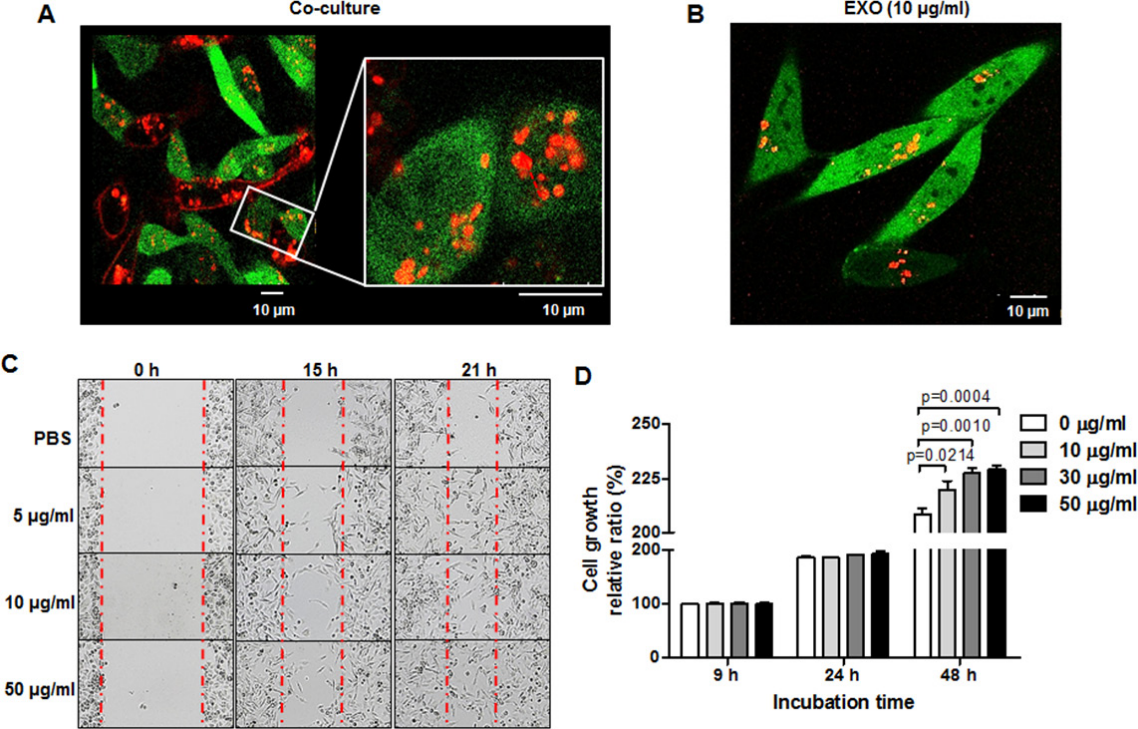
TNBC cell migration and proliferation is enhanced by TNBC cell–derived exosomes (**A**) Confocal images of transportation of RFP-tagged exosomes in direct co-culture with MDA-MB-231/CD63-RFP cells and MDA-MB-231/GFP cells for 24 hours. (**B**) Confocal image of RFP-exosomes (EXO) taken up by MDA-MB-231/GFP cells after administration of RFP-tagged exosomes (10 µg/mL) for 24 hours. (**C**)Wound-healing assay in MDA-MB-231 cells treated with RFP-tagged exosomes (5–10 µg/mL) or PBS for 15 to 21 hours. (**D**) Proliferation assay of MDA-MB-231 cells treated with RFP-tagged exosomes (10–50 µg/mL) or PBS for 24 to 48 hours.

To visualize the intercellular transfer of exosomes between breast cancer cells, we performed live cell imaging performed using confocal laser scanning microscopy. Figure 2A shows that RFP-tagged exosomes derived from MDA-MB-231/CD63-RFP cells were translocated into MDA-MB-231/GFP cells under direct co-culture with MDA-MB-231/CD63-RFP cells for 24 hours (Figure 2A). Figure 2B depicts the internalization of RFP-tagged exosomes in MDA-MB-231/GFP cells 24 hours after treatment with 10 µg/mL of RFP-tagged exosomes isolated from MDA-MB-231/CD63-RFP cells.

We investigated the effects of exosomes on MDA-MB-231 cell migration and proliferation. MDA-MB-231 cell migration was promoted by the administration of their secreted RFP-tagged exosomes (5–50 µg/mL) in a time-dependent manner (Figure 2C). MDA-MB-231 cell proliferation at 48 hours was enhanced in the RFP-tagged exosome–treated group (219.4 ± 2.538, *P* = 0.0214; 227.4 ± 1.466, *P* = 0.001; and 229.2 ± 0.984, *P* = 0.0004 at 10, 30, and 50 µg/mL, respectively) compared with the control group (208.4 ± 1.624) (Figure 2D). To test the effect of exosome removal rather than administration on MDA-MB-231 cell growth, we treated the exosome-releasing inhibitor 5-(N, N-Dimethyl) amiloride hydrochloride (DMA, 15 µM) or the exosome-uptake inhibitor methyl-β-cyclodextrin (MβCD, 10 mM) for 24 hours and 48 hours. We observed the suppression of MDA-MB-231 cell growth in the presence of DMA or MβCD (Supplementary Figure 1).

### TBNC cell–derived exosomes promote the migration and M2 polarization of macrophages *in vitro* and *in vivo*

**Figure 3:**
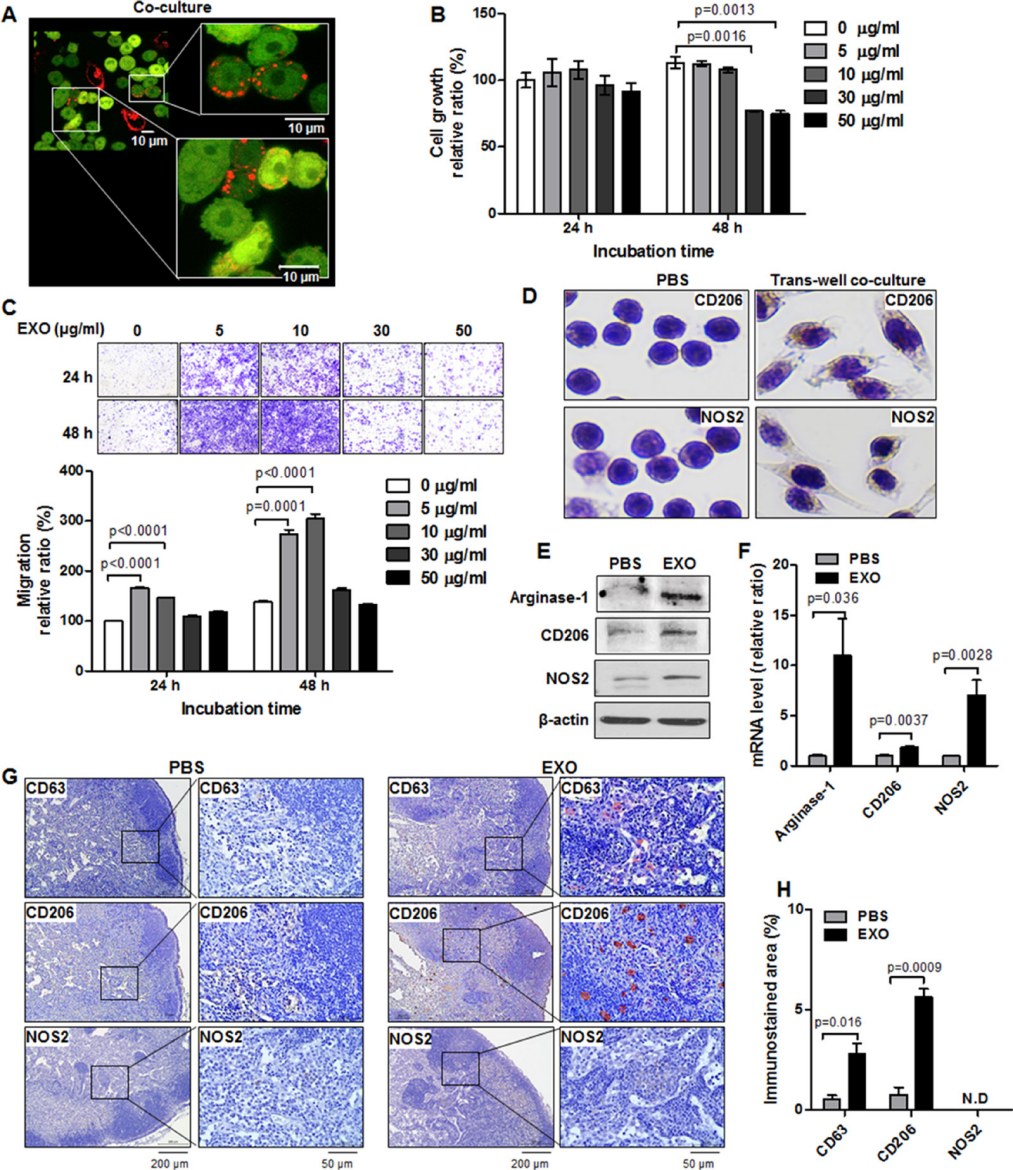
Induction of M1/M2 polarization by TNBC cell–derived exosomes *in vitro* and *in vivo* (**A**) Confocal images of RFP-tagged exosome transportation in direct co-culture with MDA-MB-231/CD63-RFP cells and RAW264.7/GFP cells for 24 hours. (**B**) Proliferation assay in RAW264.7 cells treated with RFP-tagged exosomes (30 or 50 µg/mL) or PBS for 24 to 48 hours. (**C**) Trans-well migration assay in RAW264.7 cells treated with RFP-tagged exosomes (EXO, 5–10 µg/mL) or PBS for 24 to 48 hours. (**D**) Immunostaining of CD206 and NOS2 in RAW264.7 cells cultivated with MDA-MB-231/CD63-RFP cells in the trans-well system for 24 hours. (**E** and **F**) Western blot and real-time RT-PCR of arginase-1, CD206, and NOS2 in RAW264.7 cells administered RFP-tagged exosomes (10 µg/mL) or PBS for 24 to 48 hours. (**G**) Immunostaining images of CD63, CD206, and NOS2 in axillary LNs removed from mice at 3 hours after intravenous injection of RFP-tagged exosomes (100 µg) or PBS. (**H**) Quantitative immunostained area (mean ± S.E.) of CD63, CD206, and NOS2. ND indicates no detection.

To image exosomes transferred from cancer cells to macrophages, we performed direct co-culture of MDA-MB-231/CD63-RFP cells and macrophage RAW264.7 cells. Live images of RFP-tagged exosomes taken up by RAW264.7 cells were captured by confocal laser scanning microscopy. As shown in Figure 3A, most of the RAW264.7/GFP cells took up RFP-tagged exosomes and exhibited slightly elongated morphology after co-culture with MDA-MB-231/CD63-RFP cells for 24 hours.

Macrophage proliferation and migration, which promote the immune response, were evaluated by MTT assay and trans-well migration assay. Macrophage growth is not suppressed after treatment with 5 or 10 µg/mL exosomes, implying that cancer-derived exosomes exert low cytotoxic effects, but the administration of 30 or 50 µg/mL exosomes for 48 hours reduced macrophage growth (76.93 ± 0.53, *P* = 0.0013 or 74.80 ± 2.37, *P* = 0.0016) (Figure 3B). Figure 3C is an image of crystal violet staining of migrated RAW264.7 cells treated with different doses of RFP-tagged exosomes (5–50 µg/mL). The administration of low doses (5–10 µg/mL) of RFP-tagged exosomes for 24 hours or 48 hours resulted in an approximate 1.5-fold to 2-fold increase in macrophage migration (166.14 ± 1.73 or 146.31 ± 1.05 versus 100.0 ± 0.73, *P* < 0.0001, 24 hours and 273.82 ± 8.52 or 304.49 ± 9.61 versus 137.74 ± 2.14, *P* ≤ 0.0001, 48 hours) compared with untreated cells, whereas treatment with 30 to 50 µg/mL RFP-tagged exosomes exerted the cytotoxic effect as assessed by trypan blue assay and flow cytometric analysis with propidium iodide DNA staining (Supplementary Figure 2), and did not influence macrophage migration (Figure 3C), indicating that the growth-inhibitory and low migration-promoting effects of 30 to 50 µg/mL exosomes are caused by the cytotoxicity.

To evaluate M1 and M2 polarization of RAW264.7 cells treated with TNBC cell–derived exosomes, we investigated the expression of M1 (NOS2) and M2 (CD206, arginase-1) markers. In trans-well co-culture with RAW264.7 and MDA-MB-231/CD63-RFP cells, we observed that CD206 staining intensity in RAW264.7 cells increased compared with NOS2 cells (Figure 3D). After 24 to 48 hours of treatment with 10 µg/mL RFP-tagged exosomes, which does not cause cytotoxic effects in RAW264.7 cells, arginase-1, CD206, and NOS2 protein levels increased as compared with those of PBS-treated RAW264.7 cells (Figure 3E). In quantitative RT-PCR analysis, the administration of RFP-tagged exosomes (10 µg/mL) for 24 hours resulted in an increase in mRNAs expression of arginase-1 (11.0 ± 0.3.67, *P =* 0.036), CD206 (1.89 ± 0.08, *P* = 0.0037), and NOS2 (7.29 ± 1.53, *P* = 0.0028) compared with those of PBS-treated RAW264.7 cells (Figure 3F). In evaluation of additional M2 markers FIZZ-1 and YM-1in RAW264.7, the administration of RFP-tagged exosomes of MDA-MB-231 cells increased FIZZ-1 mRNA, but YM-1 mRNA expression was not detected (Supplementary Figure 3). We further evaluated the macrophage polarization markers of other breast cancer-derived exosomes, such as MCF-7, Hs578T, and HCC-38. NOS2 and arginiase-1 levels increased in MCF-7, Hs578T, and HCC-38 exosome-treated RAW264.7 cells relative to PBS-treated cells, and CD206, FIZZ-1, and YM-1 mRNA expressions were undetectable or decreased in MCF-7, Hs578T, and HCC-38 exosome–treated RAW264.7 cells (Supplementary Figure 3).

To determine the *in vivo* fate of TNBC cell–derived exosomes after intravenous injection, we labeled RFP-tagged exosomes (100 µg) with a lipid-associated fluorescent dye, administered into tail veins of non–tumor-bearing mice and monitored using *in vivo* and *ex vivo* optical imaging. The DiD signal was detected in the liver area of the mice 3 hours after intravenous injection and disappeared at 48 hours (Supplementary Figure 4A). *Ex vivo* DiD signals were strongest in liver and spleen tissues and were not detected in the other tissues (Supplementary Figure 4B). A large number of exosomes was captured in the liver and spleen after intravenous injection.

We examined *in vivo* M1 or M2 polarization in axillary LNs of non–tumor-bearing mice injected with TNBC cell–derived exosomes. M1 polarization marker (NOS2) expression was not detected, whereas M2 polarization marker (CD206) expression was detected in axillary LNs exhibiting CD63 (exosomal marker) at 3 hours after the injection with RFP-tagged exosomes (Figure 3G). The CD63-positive areas in PBS-injected LNs and exosome-injected LNs were 0.57% ± 0.17% and 2.80% ± 0.53%, respectively. The CD206-positive areas in PBS-injected LNs and exosome-injected LNs were 0.77% ± 0.38% and 5.65% ± 0.41%, respectively.

### Intravenous injection of TNBC cell–derived exosomes promotes axillary LN metastasis in orthotopic breast cancer models

**Figure 4:**
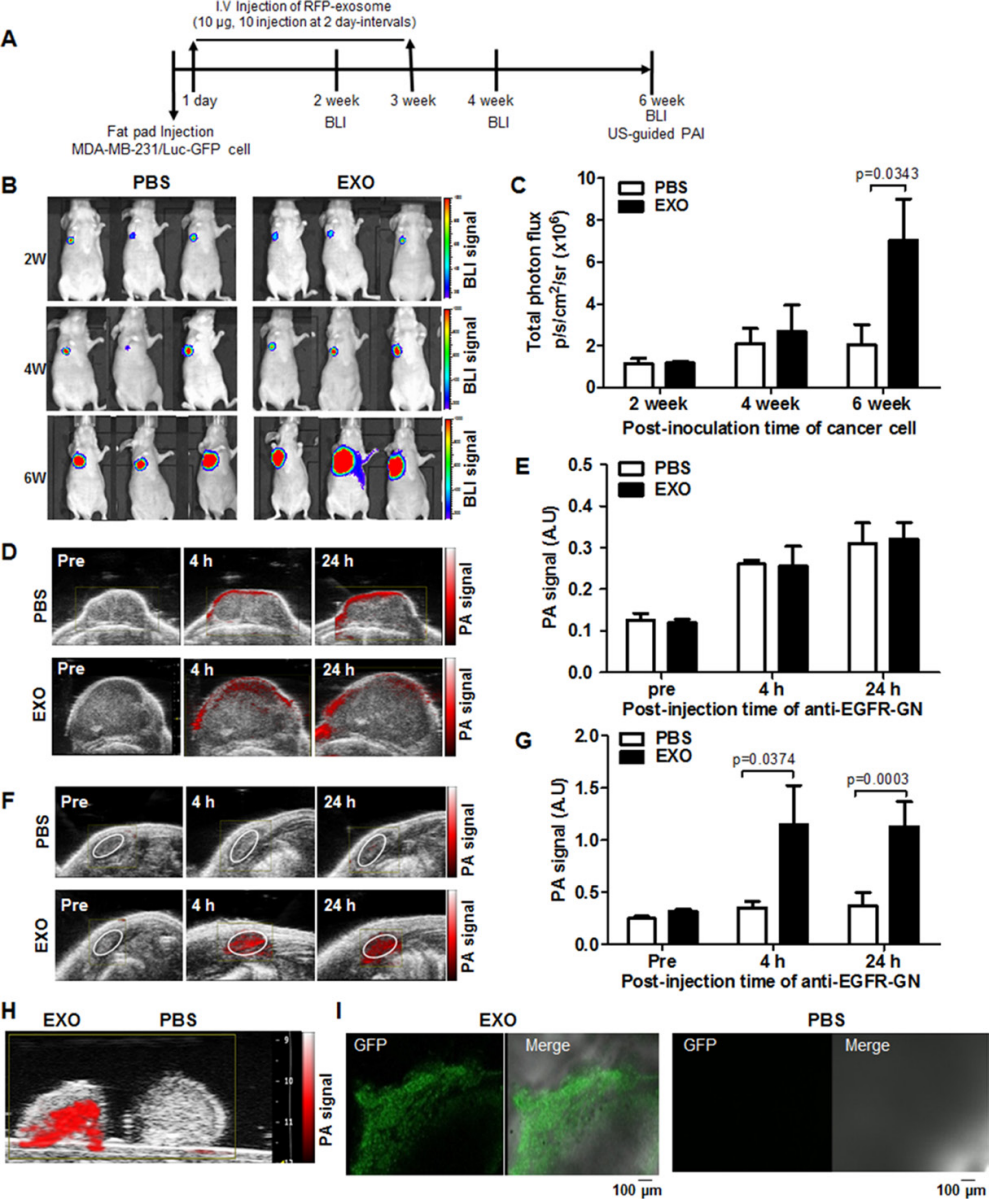
Noninvasive BLI and US-guided PAI of primary tumor growth and axillary LN metastasis promoted by cancer cell–derived exosomes in TNBC models (**A**) A flowchart depicting the experimental design in an orthotropic tumor model. (**B**) Representative BLI of primary tumors of mice intravenously injected with PBS or RFP-tagged exosomes (EXO, 10 µg, 10 injections at 2 day-intervals) at 2, 4, and 6 weeks after fat pad injection with MDA-MB-231/Luc-GFP cells. (**C**) Total photon flux (mean ± S.E.) measured from primary tumors. (**D** and **F**) Representative US-guided PAI of primary tumor and axillary LNs of mice intravenously injected with PBS or RFP-tagged exosomes before and 4 hours and 24 hours after intratumor injection of anti–EGFR-GN (7.7 mg/kg). (**E** and **G**) PA signals (mean ± S.E.) measured from primary tumors and axillary LNs. (**H** and **I**) Representative *ex vivo* US-guided PAI and GFP confocal images of axillary LNs isolated from mice intravenously injected with PBS or RFP-tagged exosomes.

To investigate the effect of cancer-derived exosomes on breast tumor progression and metastasis, we noninvasively monitored primary tumor growth and LN metastasis in mice after intravenous injection of RFP-tagged exosomes (10 µg, 10 injections at 2-day intervals) using BLI and US-guided PAI (Figure 4A). BLI signals gradually increased in both PBS-injected and exosome-injected tumors (Figure 4B), and quantitative photon fluxes of primary tumors increased in exosome-injected mice (7.03 ± 1.97 × 10^6^ p/s/cm^2^/sr) compared with PBS-injected mice (2.06 ± 0.95 × 10^6^ p/s/cm^2^/sr) at 6 weeks after inoculation (*P* = 0.0343, Figure 4C). The tumor volumes of exosome-injected mice (300.67 ± 34.06 mm^2^) increased compared with PBS-injected mice (169.99 ± 47.61 mm^2^) at 6 weeks (*P* = 0.0496, Supplementary Figure 5A). We noted no differences in average tumor volumes and BLI signals between PBS-injected mice and exosome-injected mice at 4 weeks (Figure 4C and Supplementary Figure 5A). However, at 4 weeks after inoculation, BLI signal (9.46 ± 0.1 × 10^4^ p/s/cm^2^/sr) in the area of axillary LN metastasis can be detected in 2 of 7 exosome-injected mice, whereas the BLI signal was not detected in the axillary LNs of PBS-injected mice (Supplementary Figure 5B and 5C).

Anti–EGFR-GNs are an active PAI contrast agent that selectively binds to EGFR-positive tumor cells in primary tumor mass and regional metastatic LN [23]. To noninvasively detect EGFR-positive tumors with axillary LN metastasis in mice after the intratumoral injection of anti-EGFR-GNs, we performed US-guided PAI. The representative US-guided PAIs of PBS-injected or exosome-injected primary tumors before and 4 hours and 24 hours after the injection of anti–EGFR-GNs (7.7 mg/kg) into primary tumors at 6 weeks are presented in Figure 4D. We detected strong PAI signals on the periphery of primary tumors in PBS-injected or exosome-injected mice up to 24 hours after injection of anti–EGFR-GN, we noted no differences in average PA signals in the primary tumor area of PBS-injected and exosome-injected mice (Figure 4E). The serial follow-up US-guided PAIs of axillary LNs in PBS-injected or exosome-injected mice are presented in Figure 4F. Average PAI signals in axillary LNs of exosome-injected mice at 4 hours and 24 hours increased compared with PBS-injected mice (1.46 ± 0.45 AU versus 0.31 ± 0.05 AU, *P* = 0.037 and 1.43 ± 0.19 AU versus 0.25 ± 0.03 AU, *P* = 0.0003) (Figure 4G). Based on *in vivo* US-guided PAI analysis, enhanced PAI signals in the axillary LNs in 5 of 7 exosome-injected mice and in 1 of 6 PBS-injected mice indicated that axillary LN metastasis increased on cancer exosome injection. *Ex vivo* results of US-guided PAI and confocal GFP fluorescence microscopy images of axillary LN dissections were consistent with *in vivo* imaging analysis (Figure 4H and 4I).

### Histological analysis of axillary LNs correlated with anti–EGFR-enhanced PAI signals in PBS-injected or exosome-injected mice

**Figure 5:**
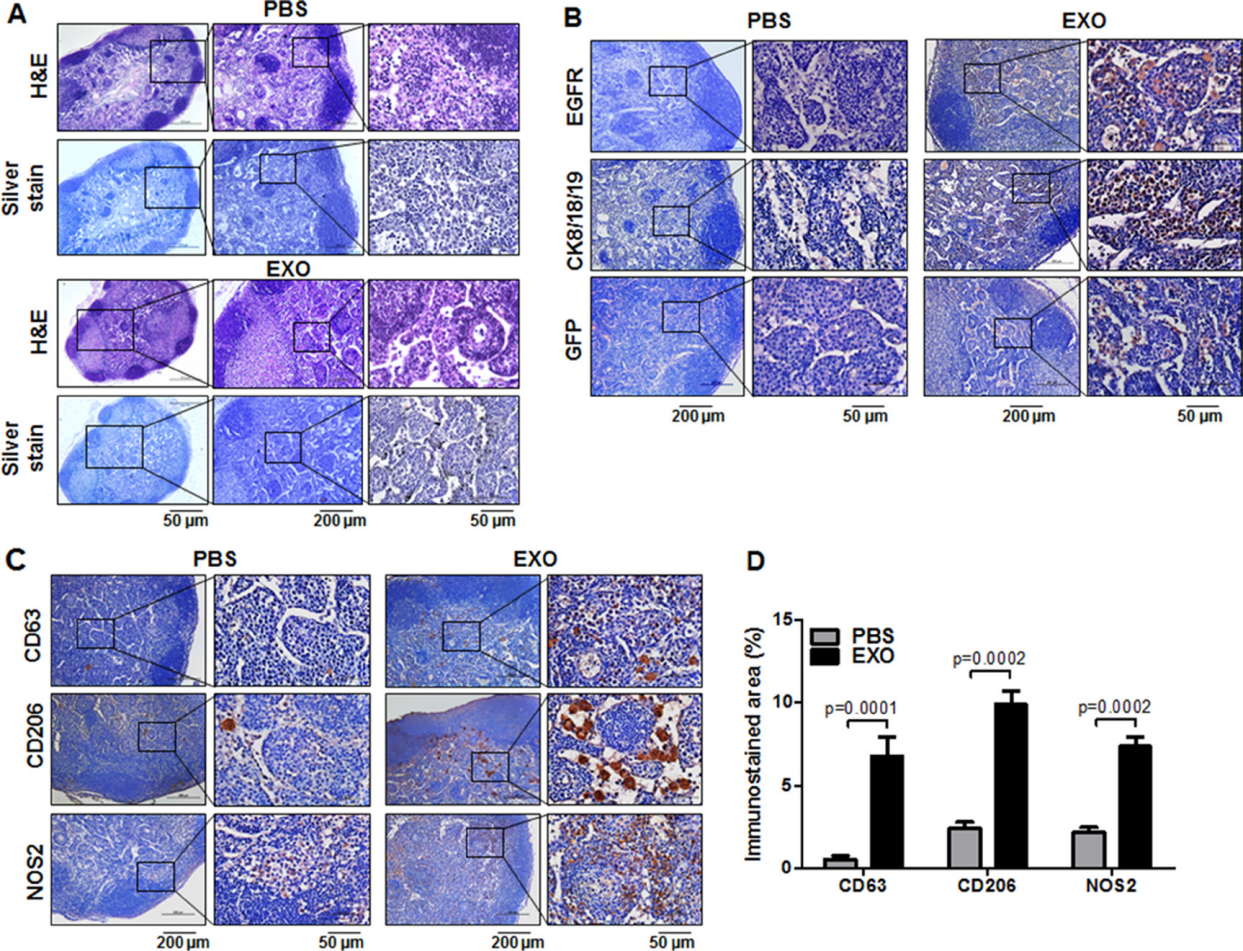
Histological analysis of axillary LN metastasis promoted by cancer cell–derived exosomes in TNBC models After follow-up of tumor growth and axillary LN metastasis by use of biweekly BLI and US-guided PAI by intratumor injection with anti–EGFR-GNs (7.7 mg/kg), axillary LNs were isolated from tumor-bearing mice injected with RFP-tagged exosomes (EXO) or PBS at 6 weeks. (**A**) H&E and silver staining images for the investigation of anti–EGFR-GNs accumulation in axillary LNs. (**B**). Immunostaining images of EGFR, CK18/8/19, and GFP for the evaluation of metastasis in axillary LNs. (**C**) Immunostaining images of CD206 and NOS2 for the evaluation of macrophage M2/M1 polarization in axillary LNs. Immunostaining images of CD63 for the analysis of cancer-derived exosome distribution in axillary LNs. (**D**) Quantitative immunostained area (mean ± S.E.) of CD63, CD206, and NOS2.

To verify the presence of anti–EGFR-GNs, which selectively target MDA-MB-231 cells, we performed silver staining in axillary LNs of tumor-bearing mice. As shown in Figure 5A, large numbers of anti–EGFR-GNs accumulated in the cortex of axillary LNs in 5 of 7 exosome-injected mice, but we observed anti–EGFR-GNs accumulation in axillary LNs in only 1 of 6 PBS-injected mice. The silver staining results correlate with *in vivo* and *ex vivo* US-guided PAI results (Table 1). The silver staining results were positive in all cases with *in vivo* PAI signals over 0.8 AU.

**Table 1:** Correlationship between histological analysis of CK8/18/19, EGFR, silver staining, and anti-EGFR-enhanced photoacoustic imaging (PAI) signal analysis in each axillary LN of PBS- or exosome-injected mice

No	PBS-injected group (*n =* 6)					Exosome-injected group (*n =* 7)				
	LN metastasis	CK8/18/19 (%)	EGFR (%)	Silver staining	PAI (AU)	LN metastasis	CK8/18/19 (%)	EGFR (%)	Silver staining	PAI (AU)
1	Absent	0	0	N.D.	0.31	Present	8.54	9.50	+	1.54
2	Absent	0	0	N.D.	0.12	Present	4.89	2.96	N.D.	0.37
3	Present	7.72	7.78	+	0.98	Present	13.79	9.69	+	0.84
4	Absent	0	0	N.D.	0.28	Present	13.23	11.99	+	1.43
5	Present	4.32	3.34	N.D.	0.24	Present	7.4	7.39	+	2.03
6	Absent	0	0	N.D.	0.28	Absent	0	0	N.D.	0.38
7	NA	NA	NA	NA	NA	Present	7.96	6.75	+	1.33

N.D.; no detection, +: detection, AU; arbitrary unit, NA; not applicable.

To confirm MDA-MB-231/Luc-GFP cell metastases in axillary LNs, we performed immunostainings for EGFR, cytokeratin 8/18/19 (CK8/18/19), and GFP. We observed strong immunostainings for EGFR, CK8/18/19, and GFP in the cortex zone of all axillary LNs of the exosome-injected group, but we observed minimal or no staining in most axillary LNs of the PBS-injected group (Figure 5B). Based on CK8/18/19 and EGFR immunostaining analysis, we detected axillary LN metastases in 6 of 7 exosome-injected mice and 2 of 6 PBS-injected mice. We investigated the relation among histological analysis of CK8/18/19, EGFR, silver staining, and PAI signal analysis at 24 hours after injection of anti–EGFR-GNs in each axillary LN of PBS-injected (*n* = 6) or exosome-injected (*n* = 7) mouse (Table 1). The area of CK8/18/19 and EGFR immunostaining in metastatic axillary LNs included approximately 3% to 8% in 2 PBS-injected mice and approximately 3% to 14% in 5 exosome-injected mice. PA signals in axillary LNs correlated with EGFR staining in both groups (Spearman γ = 0.7414, *P* = 0.0037). In addition, PA signals from 8 metastatic axillary LNs (1.199 ± 0.217 AU) were increased compared with 5 non-metastatic LNs (0.290 ± 0.038 AU, *P* = 0.0029). The immunostaining area of CK8/18/19 and EGFR was less than 5% in axillary LNs of 1 PBS-injected mouse and 2 exosome-injected mice, but we did not detect anti–EGFR-GN-enhanced PA signals and silver stain in these axillary LNs.

### TNBC cell exosome–promoted axillary LN metastasis is associated with an increased ratio of M2/M1 polarized macrophages

We examined the presence of TNBC cell–derived exosomes using antibodies for the detection of human CD63 in axillary LNs of tumor-bearing mice (Figure 5C). We observed increased CD63 expression in the cortex area of LNs of exosome-injected mice (6.75% ± 1.18%) compared with LNs of PBS-injected mice (0.53% ± 0.22%) (*P* = 0.0001) (Figure 5D), suggesting that cancer exosomes are involved in creating a microenvironment favorable to LN metastasis. We investigated the differential macrophage polarized phenotypes in metastatic LNs via CD206 (M2 marker) and NOS2 (M1 marker) immunostaining. As shown in Figure 5C, increased CD206 and NOS2 expression was observed in the subcapsular and cortex zone of metastatic LNs of exosomes-injected mice compared with PBS-injected mice. The macrophages in metastatic LNs exhibited mixed phenotypes, expressing both CD206 and NOS2. The CD206-positive areas in exosome-injected LNs and PBS-injected LNs were 9.90% ± 0.83% and 2.41% ± 0.38% (*P* = 0.0002), respectively (Figure 5D). The NOS2-positive areas in exosome-injected LNs and PBS-injected LNs were 7.35% ± 0.58% and 2.15% ± 0.33% (*P* = 0.0002), respectively (Figure 5D). The ratio of M2 (CD206) to M1 (NOS2) macrophages increased by approximately 1.5-fold in exosome-injected LNs compared with PBS-injected LNs. This finding indicates that crosstalk between TNBC cell–derived exosomes and M2 polarized macrophages was more extensive in promoting axillary LN metastasis.

## DISCUSSION

We demonstrate that aggressive TNBC cell (MDA-MB-231)–derived exosomes function as intracellular links between cancer cells and macrophages. We demonstrated that TNBC cell–derived exosomes are a factor in the induction of M2-type macrophage polarization (upregulation of CD206 and arginase-1) to the benefit of breast cancer cells *in vitro* and *in vivo*, supporting enhanced tumor growth and axillary LN metastasis in an orthotopic TNBC model. We observed increases in primary tumor growth and axillary LN metastasis in orthotopic TNBC mice intravenously administered TNBC cell–derived exosomes using noninvasive BLI and US-guided PAI. Our results provide the evidence of an activator of TNBC cell–derived exosomes capable of accelerating tumor progression and LN metastatic dissemination in TNBC. Our findings are consistent with research that determined exosomes secreted from the other types of tumor cells, including pancreatic, ovarian, and gastric cancer cells, appear to promote metastasis [17–22].

Noninvasive imaging of exosomes expressing reporter proteins enables real-time tracking of intracellular transfer of exosomes. We established a TNBC cell line (MDA-MB-231/CD63-RFP cells) that produces RFP-tagged exosomes by introduction of CD63-RFP fusion genes to enable the noninvasive monitoring of exosome transfer between TNBC cells and macrophages (RAW264.7). Co-culture or exosome administration enabled noninvasive monitoring of the intracellular transfer of RFP-tagged exosomes between cancer cells and macrophages, which demonstrated that TNBC cell–derived exosome transfer is dynamic. We visualized the exosomes from cells that harbor the Glu-lactadherin construct, a reporter protein that emits bioluminescence, *in vivo* after intravenous injection [24], but we failed to visualize RFP-tagged exosomes after intravenous injection. Compared with bioluminescent Glu-lactadherin imaging, fluorescent RFP imaging is less sensitive and is not appropriate for *in vivo* application to monitor exosomes. However, we noninvasively monitored the intravenously injected exosomes using the DiD-labeling method. DiD-labeled exosome imaging results demonstrated that TNBC cell–derived exosomes undergo simultaneous hematogenous and lymphatic spread, producing metastatic deposits in LN, liver, and lungs. Noninvasive imaging tools for direct exosome tracking by optical reporter can be used to explore the pathophysiological function of exosomes in organs during tumor progression.

We investigated anti–EGFR-enhanced signal analysis of PAI in each axillary LN of PBS-injected or exosome-injected mice. PAI integrates with the clinical US imaging system, US-guided PAI, to simultaneously provide structural, functional, and molecular information at clinically relevant penetration depths by use of exogenous contrast agents. This technique was introduced as an approach for more sensitive and accurate detection of tumor and LN metastases *in vivo* [25–27]. We previously reported that anti–EGFR-GNs-enhanced PAI is more sensitive than bioluminescence imaging for the detection of axillary LN micrometastasis [23]. The accuracy of PAI-detected LN metastasis was significant in 75% of mice (6 of 8 mice) based on correlation with histological analysis. In the present study, PAI detects axillary LN metastasis only when the area exhibits more than 3% EGFR immunostaining. Although these imaging approaches have demonstrated potential in providing useful morphological and functional information to detect LN metastasis, an imaging technique that can accurately detect LN micrometastases in real-time is needed.

Cancer cells communicate with neighboring cells via exosomes or other pathways to induce primary tumor growth and metastatic outgrowth. Macrophages exhibited plasticity via the M1 to M2 switch at various steps to enhance tumor initiation, growth, and metastasis. The presence of M2-type macrophages is clinically associated with poor prognosis in various types of cancers [28], which suggests that macrophages induce metastatic development by distinct cellular interactions within metastatic sites. Compared with ER-positive breast cancer cells, aggressive TNBC cells exert macrophage immunomodulation and induce M2-like polarization and inflammatory cytokine production [16, 17]. Increased numbers of M2-type macrophages are found in metastatic LNs and are considered an index to predict LN metastasis [14, 15, 29]. Consistent with the aforementioned studies, we observed TNBC-derived exosome–induced macrophage programming and upregulation of both M1 marker (NOS2) and M2 marker (CD206, arginase-1) in cultured macrophages taking up RFP-tagged exosomes and metastatic LNs of mice injected with RFP-tagged exosomes. After exosome administration, NOS2 expression was detected in the axillary LNs of tumor-bearing mice but not in the axillary of healthy mice without tumors, and upregulated in the metastatic LNs. Such differences might be caused by the number of cancer-derived exosomes required to induce NOS2 expression. Thus, the exosomes continuously released from metastatic LNs and additional exosome administration was enough to induce NOS2 expression in tumor-bearing mice. Our finding is consistent with a previous study that reports the association with high NOS2 expression and metastasis in breast cancer patients [30]. Metastatic TNBC cells assessed by EGFR, CK8/18/19, or GFP immunostaining were localized in the cortex zones of the axillary LNs. Many CD206-positive M2-type macrophages were also localized in the cortex zone of axillary LNs in RFP-tagged exosome–injected mice. These results suggest that the increase in M2-type macrophages promotes LN metastasis and might be an attractive index for LN metastasis in TNBC patients.

Cancer exosomes are carriers of pro-tumorigenic molecules, such as protein, mRNA, microRNAs, and lipids, that induce macrophage polarization, thereby promoting cancer growth and metastasis [31]. Studies have revealed the exosomal molecules associated with macrophage immunomodulation in diverse cancers. Exosomes derived from ovarian cancer deliver microRNA-940 to induce macrophage M2 polarization [20]. Milk fat globule-EGF factor 8 (MGF-E8) upregulation in exosome proteins isolated from patients with primary and metastatic prostate cancer is associated with M2-type macrophage polarization [32]. miR-155 and miR-125b-2 transduction in pancreatic cancer cell (Panc-1)–derived exosomes converts the M2 phenotype back to the M1 phenotype [18]. Exosomal annexin II derived from breast cancer cells (MDA-MB-231) promotes breast cancer metastasis through macrophage-induced angiogenesis [33]. Wnt5a enrichment in breast cancer cell (SKBR-3)–derived exosomes is associated with macrophage-induced invasion of breast cancer cells [34]. Ovarian cancer cell (SKOV-3)–derived exosomal miR-222 induces polarization of M2-type macrophages for tumor promotion [22]. miR-19a-3p downregulation induced by conditioned medium of breast cancer cells (4T1) is associated with M2-type macrophage polarization, resulting in breast cancer progression and metastasis [35].

Our study demonstrates that TNBC-derived exosomes are a factor in tumor growth and LN metastasis through intercellular communication with macrophages. However, the crucial contents of breast cancer exosomes are still not fully elucidated, and considerable research is needed to understand the role of TNBC cell–derived exosomes in macrophage polarization and breast cancer progression. Our next study will focus on identifying the crucial molecular components of TNBC cell–derived exosomes and deciphering the molecular mechanisms involved in macrophage polarization and LN metastasis.

## MATERIALS AND METHODS

### Cell culture and antibody reagents

The human TNBC cell line (MDA-MB-231) and murine macrophage (Raw264.7) were obtained from the Korean Cell Line Bank (Seoul, Korea). All cells were grown in Roswell Park Memorial Institute (RPMI) 1640 medium (WelGENE, Daegu, Korea) containing 10% fetal bovine serum (FBS) and supplemented with a 1% antibiotic solution containing penicillin and streptomycin (Thermo Fisher Scientific, Waltham, MA, USA). Cells were cultured in a 5% CO_2_ incubator at 37°C. The primary antibodies used in this study were anti-cytokeratin 8/18/19 antibody, anti-GFP antibody, anti-RFP antibody, anti-CD63 antibody, anti-ALIX antibody, and anti-calnexin antibody, purchased from Abcam (Cambridge, MA, USA). Anti-NOS2 antibody, anti-CD206 antibody, anti-arginase-1 antibody, and anti-GAPDH antibody were purchased from Santa Cruz Biotechnology (Dallas, Texas, USA). Anti-β-actin antibody was purchased from Sigma (St. Louis, MO, USA).

### Exosome isolation and characterization

A stable MDA-MB-231/CD63-RFP cell line overexpressing the exosomal CD63-RFP fusion protein was generated for monitoring RFP-tagged exosomes. Conditioned media were obtained from MDA-MB-231/CD63-RFP grown at sub-confluence for 3 to 4 days in growth media containing serum depleted of bovine exosomes (Gibco Laboratories, Carlsbad, CA, USA). For isolation of exosomes from conditioned media, the Exo-spin Exosome Purification kit was used according to the manufacturer’s instructions (Cell Guidance Systems, Cambridge, UK). Purified exosomes were then stored at −80°C until use.

### Imaging of exosome transfer in cultured cells

For imaging of RFP-tagged exosome transfer in co-culture, MDA-MB-231/GFP (green fluorescence protein-tranduced MDA-MB-231) cells, RAW264.7/GFP (GFP-transduced RAW264.7) cells, and MDA-MB-231/CD63-RFP cells were mixed (1:1) and cultured for 72 hours. Real-time imaging of RFP-tagged exosome transfer from MDA-MB-231/CD63-RFP cells to MDA-MB-231/GFP cells or RAW264.7/GFP cells was performed by use of a laser scanning confocal microscope (Leica, Wetzlar, Germany). To visualize RFP-tagged exosome uptake by MDA-MB-231/GFP cells or RAW264.7/GFP cells, isolated exosomes were resuspended in phosphate buffered saline (PBS) and quantified by use of the Pierce micro-BCA protein assay kit (ThermoScientific, Waltham, MA, USA). After administration of 10 µg of exosomes into cultured MDA-MB-231/GFP cells or RAW264.7/GFP cells, the cellular uptake of RFP-tagged exosomes was monitored by use of a laser scanning confocal microscope (Leica, Wetzlar, Germany).

### Wound-healing assay

To assess the effect of exosomes on MDA-MB-231 cell migration, wound-healing assays were performed. MDA-MB-231 cells were seeded in a 12-well plate. When the cells formed a confluent monolayer, a scratch was generated by use of a micropipette tip, and cells were washed with PBS to remove cell debris. Purified exosomes (5–50 µg/mL) were added to MDA-MB-231 cells, and wound healing was monitored by photography. Images were obtained by use of a light microscope attached to a CCD camera (Leica, Wetzlar, Germany).

### Proliferation assay

To investigate the effect of exosomes on MDA-MB-231 and RAW264.7 cell proliferation, 1×10^4^ cells were seeded in a 96-well plate and incubated in exosome-depleted FBS media for 24 hours. The purified exosomes (5–50 µg/mL) were administered to cultured cells for 24 hours to 48 hours. The cell proliferation rate was quantified by use of the 3-(4,5-dimethylthiazol-2-yl)-2,5-diphenyltetrazolium bromide) (MTT) assay, and 10 µL of MTT reagent (5 mg/mL) were added to each well and incubated for 1 hour at 37°C. Formazan crystals were solubilized by the addition of 150 µL of dimethyl sulfoxide to each well. The optical density at 540 nm was measured by use of a microplate reader (GE Healthcare, Piscataway, NJ, USA), and the cell proliferation rate was determined.

### Trans-well migration assay

To investigate the effect of exosomes on RAW264.7 cell migration, trans-well migration assays were performed, 1 × 10^5^ Raw264.7 cells were deposited in the upper chamber of the trans-well plate with a 0.4-µm pore size (BD Bioscience, San Jose, CA, USA). The lower chamber was filled with 500 µL of serum-free medium with purified exosomes (5–50 µg/mL), and cells were incubated for 24 hours to 48 hours. Migrated cells were fixed in 4% paraformaldehyde and stained with crystal violet, and the stained images were captured by light microscope. Crystal violet from the stained membrane was finally extracted with 1% sodium dodecyl sulfate (SDS). The optical density at 550 nm was measured by use of a microplate reader (GE Healthcare, Piscataway, NJ USA), and cell migration was determined.

### Real-time RT-PCR

Total RNA was isolated by use of TRIzol Reagent (Invitrogen, Carlsbad, CA, USA) and was reverse-transcribed by use of random hexamers and Superscript III reverse transcriptase. Real-time PCR reactions were run on an ABI PRISM 7900 utilizing a SYBR Green PCR master mix (Applied Biosystems, Foster City, CA, USA) and specific primer sets for NOS2, arginase-1, CD206, FIZZ-1, and YM-1 (Supplementary Table 1). Results were analyzed by the ∆Ct method, which reflects the threshold difference between a target gene and β-actin in each sample.

### Western blot

Cells were lysed in RIPA buffer (Sigma, St. Louis, MO, USA). Proteins were separated by use of SDS-polyacrylamide gel electrophoresis (SDS-PAGE) and transferred to nitrocellulose membranes. The membranes were blocked by 5% skim milk in Tris-buffered saline containing 0.05% Tween-20 and were incubated overnight at 4°C with primary antibodies. Membranes were incubated with HRP-conjugated secondary antibodies (Santa Cruz Biotechnology, Dallas, Texas, USA). The blotted membranes were visualized by use of enhanced chemiluminescence reagents (GE Healthcare, Danderyd, Sweden).

### Immunocytochemistry

Cells were fixed in 2% paraformaldehyde and blocked by 2% bovine serum albumin. Cells were incubated with the primary antibodies for CD206 and NOS2 for 1 hour at 4°C followed by incubation with an appropriate secondary antibody for 30 minutes. Proteins were visualized with 3,3-diaminobenzidine, and hematoxylin was used as counterstain. The images were acquired by use of a microscope equipped with a CCD camera (Leica, Wetzlar, Germany).

### Animals and orthotopic breast tumor models

Female BALB/c nude mice, 5 to 6 weeks old (Orient Bio, Sungnam, Korea), were housed in the animal care facility of the Biomedical Research Institute of Seoul National University Hospital. Animal care and experimental procedures were performed in accordance with guidelines on the ethical use of animals that were approved by the Institutional Animal Care and Use Committee of Seoul National University Hospital (12-0353-C2A0). A total of 19 female Balb/c nude mice were used for BLI and US-guided PAI and histological studies. For exploration of the microenvironment modifications of axillary LN by tumor exosomes, healthy mice were assigned to two groups: PBS (*n* = 3) and exosomes (*n* = 3). The purified RFP-tagged exosomes (100 µg) were labeled with DiD and intravenously injected into the tail vein. Noninvasive *in vivo* and *ex vivo* monitoring of DiD-labeled RFP-tagged exosomes by use of the IVIS luminal II system (Caliper, Hopkinton, MA, USA) and LN histological analysis were performed 3 hours and 48 hours after exosome injection.

Approximately 1 × 10^6^ viable cells were injected into the right fat pad of the first mammary gland. Tumor volume was measured with digital calipers and US imaging by use of a modified ellipsoidal formula for volume (volume = 1/2 [length×width^2^]) [36]. Tumor-bearing mice injected with MDA-MB-231/Luc-GFP (luciferase and green fluorescence protein-transduced MDA-MB-231) cells were randomly assigned to two groups: PBS (control) (*n* = 6) and exosome (*n* = 7). To investigate the function of tumor exosomes during tumor progression, purified RFP-tagged exosomes (10 µg) were administered via 10 repeated intravenous injections at 2-day intervals the day after tumor cell injection, and noninvasive imaging of tumor growth and LN metastasis followed by BLI and US-guided PAI were performed.

### BLI and US-guided PAI

*In vivo* BLI, after intraperitoneal injection of 150 ng/kg D-luciferin ( Promega, San Luis Obispo, CA, USA), was conducted on the IVIS luminal II system (Caliper, Hopkinton, MA, USA). The sum of all detected photon counts within an oval-shaped region of interest (ROI), either primary tumor or axillary LN, was quantified in units of mean photons per second per centimeter squared per steradian (p/s/cm^2^/sr) by Living Image software. Anti-EGFR-GNs were purchased from Nanopartz, Inc. (Loveland, CO, USA). Before and 4 hours to 24 hours after intratumor injection of anti-EGFR-GNs (7.7 mg/kg GN), serial follow-up US-guided PAI of primary tumors and axillary LNs was performed in B mode and PA mode on a preclinical Vevo2100 LAZR imaging system (FUJIFILM VisualSonics Inc., Toronto, Ontario, Canada) equipped with a 40-MHz linear array transducer. The laser was tuned to optical wavelengths from 750 to 850 nm with a PA signal gain of 40 dB. The relative PA signal amplitude on image slices of tumor was quantified by post-processing software tools (FUJIFILM VisualSonics Inc., Toronto, Ontario, Canada) [37].

### Histological analysis

The excised primary tumors and axillary LNs were fixed with 4% buffered formalin and embedded in paraffin blocks. Tissues were sectioned into 4-μm thick sections. Paraffin sections were deparaffinized in xylene and rehydrated in a series of graded ethanol and water solutions. For evaluation of anti-EGFR-GN accumulation, immunostaining, and immune gold-silver staining (Sigma, St. Louis, MO, USA) were performed according to the manufacturer’s protocols. Hematoxylin and eosin (H&E) staining was performed to evaluate the change in cell and tissue structure. For immunostaining, deparaffinized sections were immersed in 0.01 M sodium citrate buffer (pH 6.0) and blocked by incubation with 0.1 M NH4Cl/PBS solution and 5% normal goat serum (Gibco Laboratories, Carlsbad, CA, USA) for 30 minutes. After incubation with primary antibodies for CK8/18/19, EGFR, GFP, CD63, NOS2, and CD206 and secondary antibodies directly conjugated with HRP, the sections were visualized with a peroxidase substrate kit (SK-4100; Vector Laboratories, Burlingame, CA, USA) and counterstained with hematoxylin solution (Millipore Ltd., Darmstadt, Germany). Histological images of stained tissues were acquired by use of a microscope equipped with a CCD camera (Leica, Wetzlar, Germany). Seven fields at 40 × magnification within each section were randomly selected, and immunostained cells were quantified as the percentage of total cells in each area by Leica QWin image-analysis and image-processing software.

### Statistical analysis

Results are expressed as the mean ± standard deviation (S.D.) and statistically evaluated by use of the two-tailed unpaired *t* test. A *P*-value less than 0.05 was considered statistically significant. Statistical analyses were performed by GraphPad Prism 5.0 (GraphPad Software, Inc., La Jolla, CA, USA).

## SUPPLEMENTARY MATERIALS FIGURES AND TABLE


