# Supplementary material for: Breast cancer cell-derived exosomes and macrophage polarization are associated with lymph node metastasis
Source: Oncotarget. 2017 Dec 13;9(7):7398–410. doi: 10.18632/oncotarget.23238 (PMC5800911; doi:10.18632/oncotarget.23238)
Supplement: Supplementary file 1 [file oncotarget-09-7398-s001.pdf]

Breast cancer cell-derived exosomes and macrophage polarization are associated with lymph node metastasis

SUPPLEMENTARY MATERIALS

Supplementary Table 1: Specific primer sequence for real-time RT-PCR

Gene		Sequence (5′ -> 3′)
NOS2	Forward	CAGAGGACCCAGAGACAAGC
	Reverse	TGCTGAAACATTTCCTGTGC
Arginase-1	Forward	GGAATCTGCATGGGCAACCTGTGT
	Reverse	AGGGTCTACGTCTCGCAAGCCA
CD206	Forward	TTCGGTGGACTGTGGACGAGCA
	Reverse	ATAAGCCACCTGCCACTCCGGT
FIZZ-1	Forward	TCCAGTGAATACTGATGAGA
	Reverse	CCACTCTGGATCTCCCAAGA
YM-1	Forward	GGGCATACCTTTATCCTGAG
	Reverse	CCACTGAAGTCATCCATGTC
β-actin	Forward	TTCCTGGGCATGGAGTCCTGTGG
	Reverse	CGCCTAGAAGCATTTGCGGTGG

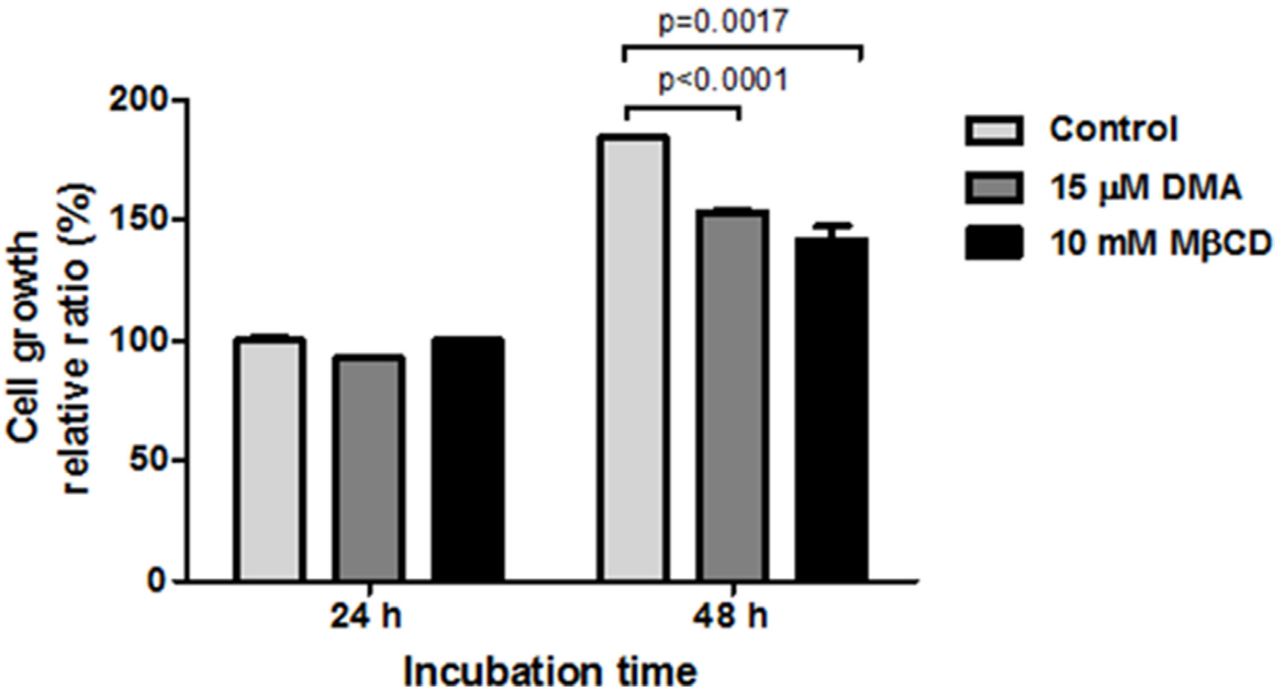

Supplementary Figure 1: TNBC cell proliferation is suppressed by blocking exosome releasing and uptake. Proliferation of MDA-MB-231 cells treated with exosome-releasing inhibitor 5-(N, N-dimethyl) amiloride hydrochloride (DMA, 15 µM) or exosome-uptake inhibitor methyl-β-cyclodextrin (MβCD, 10 mM) for 24 to 48 hours was evaluated by MTT assay.

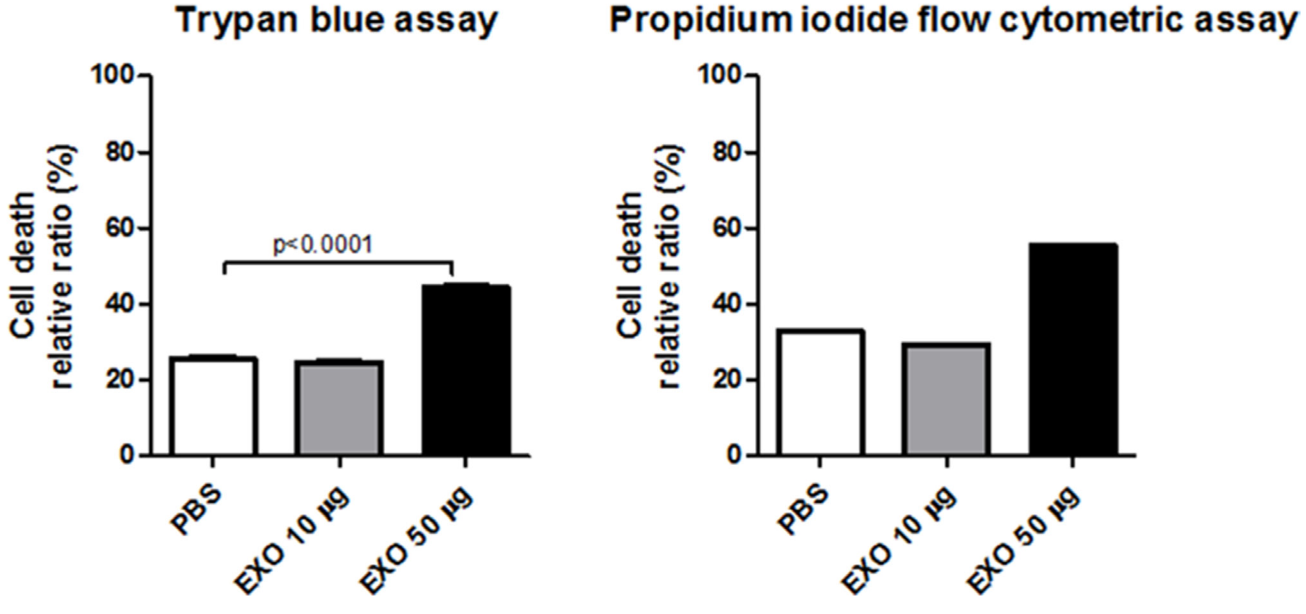

Supplementary Figure 2: High concentration of cancer-derived exosomes leads to the cytotoxicity of RAW264.7 cells. Cytotoxic effect in RAW264.7 cells treated with cancer exosomes (10 and 50 µg/mL) for 24 to 48 hours was investigated by trypan blue and propidium iodide flow cytometric assays.

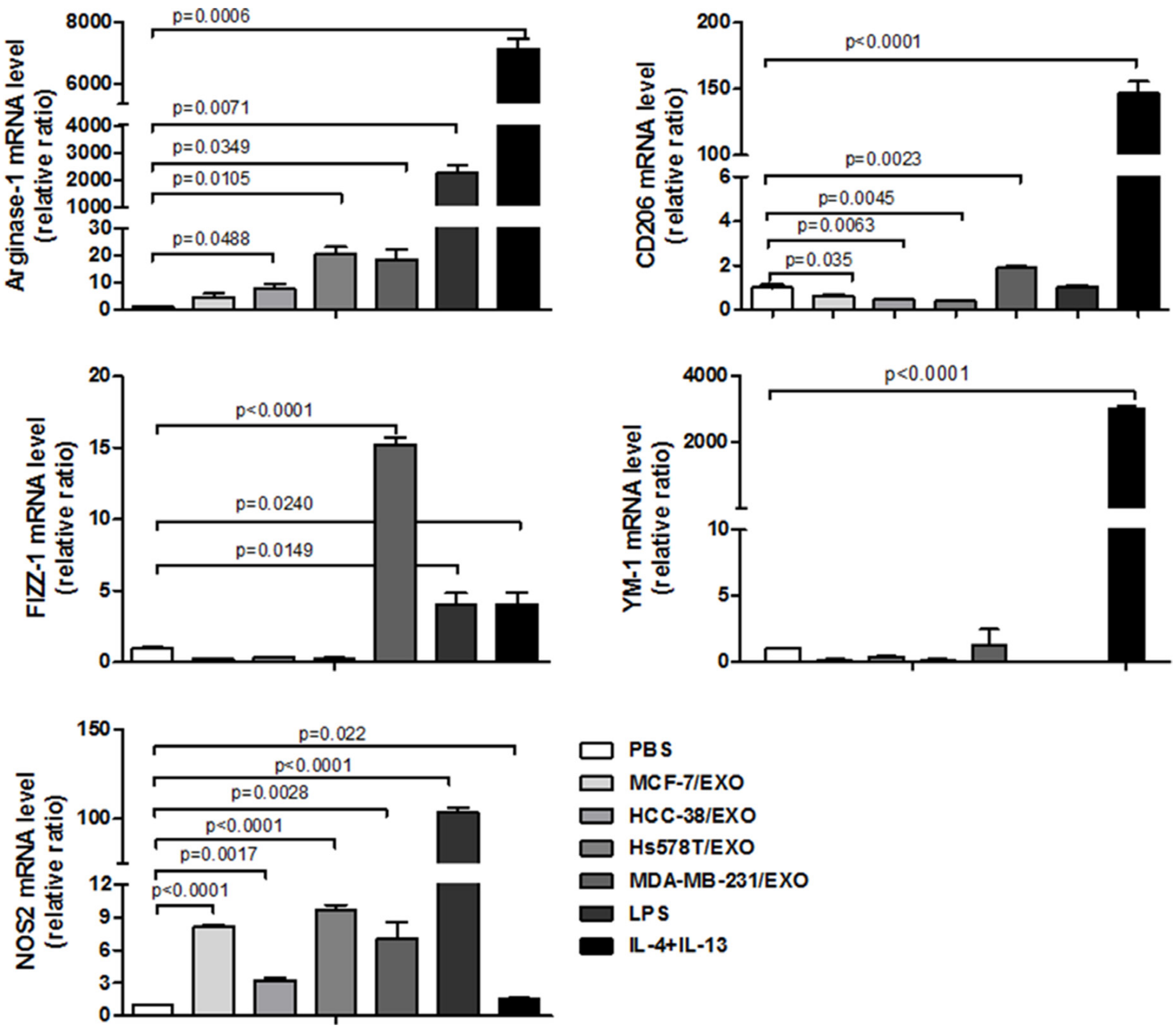

Supplementary Figure 3: Investigation of M1/M2 polarization markers in RAW264.7 cells treated with different breast cancer exosomes. Arginase-1, CD206, FIZZ-1, YM-1, and NOS2 expressions in RAW264.7 cells treated with 10 µg/mL of diverse breast cancer-exosomes (MCF-7, HCC-38, Hs578T, and MDA-MB-231), LPS (100 ng/mL) for 24 to 48 hours, or a complex of IL-4/IL-13 (20 ng/mL) for 4 days were investigated by real-time RT-PCR.

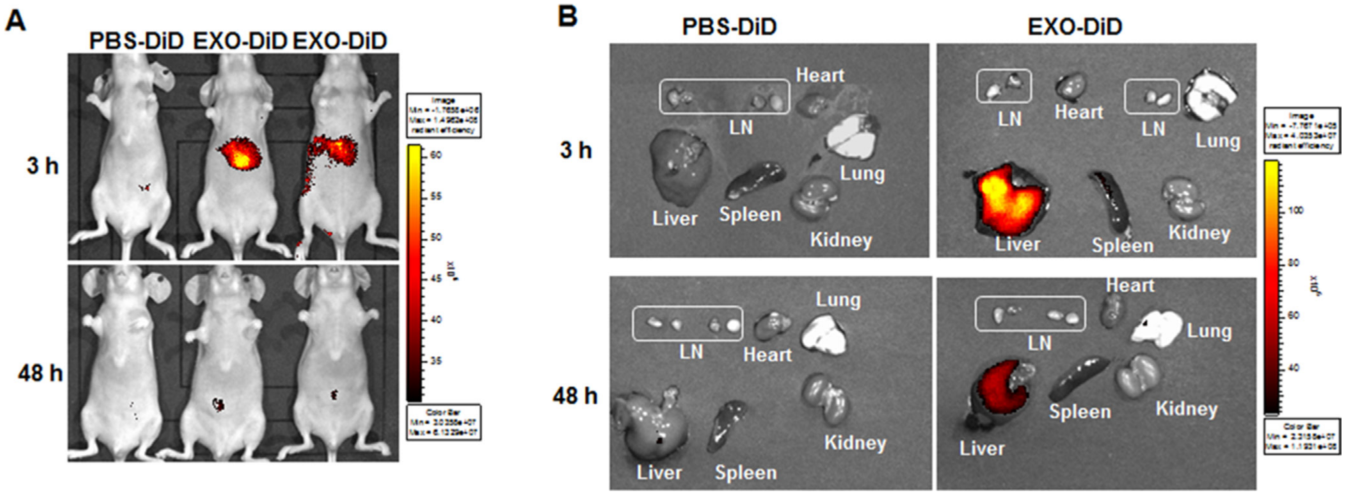

Supplementary Figure 4: Biodistribution of TNBC cell–derived exosomes. (**A**) Follow-up *in vivo* and *ex vivo* imaging of RFP-tagged exosomes (EXO) labeled with DiD in a mouse after intravenous injection. (**B**) *Ex vivo* imaging of RFP-tagged exosomes labeled with DiD in diverse tissues, including LN, heart, liver, spleen, lung, and kidney.

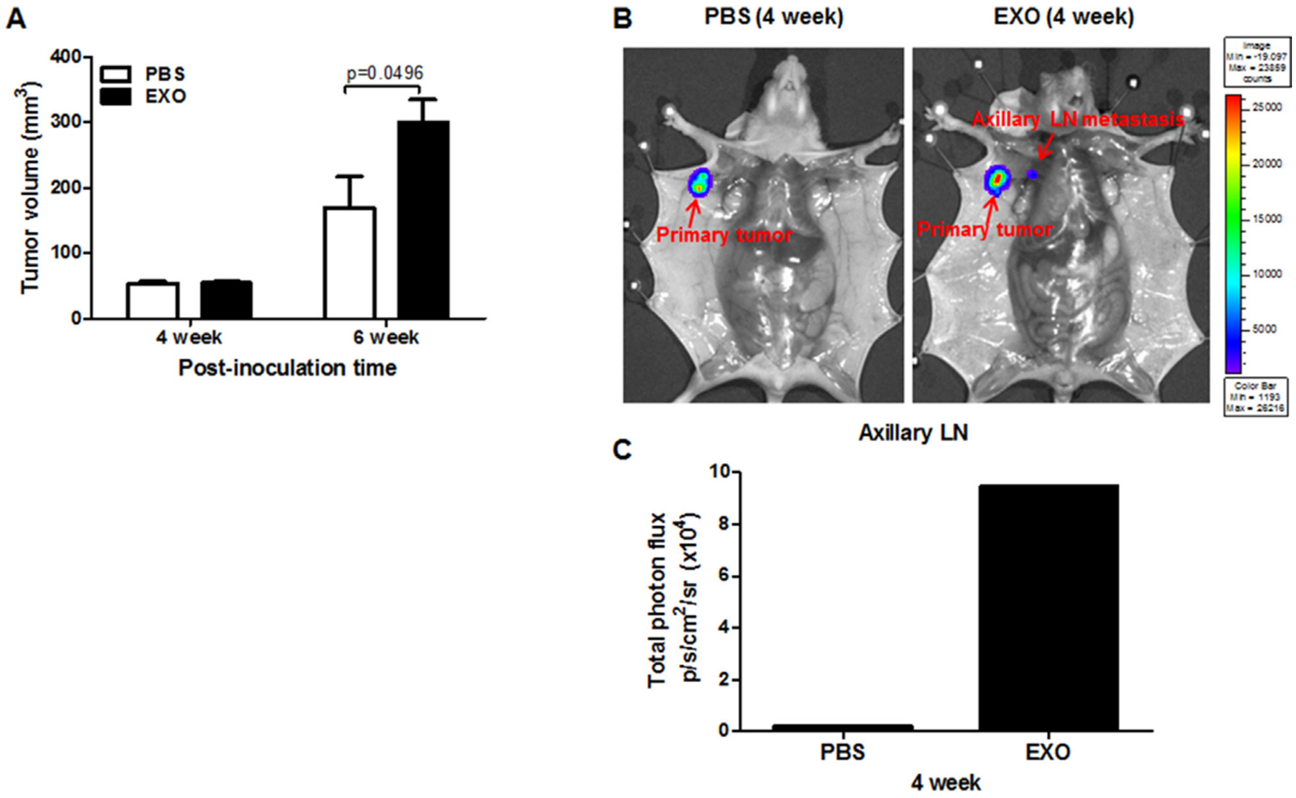

Supplementary Figure 5: Early detection of axillary LNs metastasis in xenograft tumor mice intravenously injected with TNBC cell–derived exosomes. (**A**) Xenograft tumor volume in mice administered with PBS-derived or TNBC cell–derived exosomes at 4 weeks and 6 weeks after the injection of TNBC cells. (**B** and **C**) Representative BLI and total photon flux (mean ± S.E.) of axillary LN area in tumors from mice intravenously injected with PBS or exosomes at 4 weeks.
